# Mechanical and Cytocompatibility Evaluation of UHMWPE/PCL/Bioglass^®^ Fibrous Composite for Acetabular Labrum Implant

**DOI:** 10.3390/ma12060916

**Published:** 2019-03-19

**Authors:** Adhi Anindyajati, Philip Boughton, Andrew J. Ruys

**Affiliations:** School of Aerospace, Mechanical and Mechatronic Engineering, University of Sydney, NSW 2006, Australia; philip.boughton@sydney.edu.au (P.B.); andrew.ruys@sydney.edu.au (A.J.R.)

**Keywords:** UHMWPE/PCL/Bioglass^®^, fibrous composite, labrum implant, cyclic loading, cytocompatibility

## Abstract

In this study, a fibrous composite was developed as synthetic graft for labral reconstruction treatment, comprised of ultra-high molecular weight polyethylene (UHMWPE) fabric, ultrafine fibre of polycaprolactone (PCL), and 45S5 Bioglass^®^. This experiment aimed to examine the mechanical performance and cytocompatibility of the composite. Electrospinning and a slurry dipping technique were applied for composite fabrication. To assess the mechanical performance of UHMWPE, tensile cyclic loading test was carried out. Meanwhile, cytocompatibility of the composite on fibroblastic cells was examined through a viability assay, as well as SEM images to observe cell attachment and proliferation. The mechanical test showed that the UHMWPE fabric had a mean displacement of 1.038 mm after 600 cycles, approximately 4.5 times greater resistance compared to that of natural labrum, based on data obtained from literature. A viability assay demonstrated the predominant occupation of live cells on the material surface, suggesting that the composite was able to provide a viable environment for cell growth. Meanwhile, SEM images exhibited cell adhesion and the formation of cell colonies on the material surface. These results indicated that the UHMWPE/PCL/Bioglass^®^ composite could be a promising material for labrum implants.

## 1. Introduction

Acetabular labrum is fibrocartilage tissue located in the hip joint, located between the femur and the acetabular rim [1,2]. Biomechanically, it enhances joint stability and seals the joint to protect the fluid inside [1,2,3]. Tears in this region may hinder hip joint-related activities and in the long term could even progress to osteoarthritis [4,5,6]. Therefore, it is essential to preserve labrum function. In cases of severe damage, reconstruction is often required, which involves tissue grafting [6,7,8]. However, there are several limitations associated to autografting, including source availability and the requirement of additional surgical procedures [8,9]. A synthetic graft can be an alternative approach to tackle these drawbacks.

The architecture of acetabular labrum comprises of a fibrous network [10]. To mimic the native tissue, a labrum implant is developed using fibre-based materials. These materials are made into a composite, allowing a combination of different features to be a part of one working system. To withstand mechanical loads working in the labrum area, ultra-high molecular weight polyethylene (UHMWPE) fabric is applied as an implant core or macrostructure. To facilitate cell attachment, growth, and formation of neo tissue, a biodegradable layer of aligned electrospun polycaprolactone (PCL) fibres is applied, covering the fabric. The main structure of acetabular labrum consists of highly oriented collagen fibril, hence this fine fibres layer can also mimic the natural labrum tissue [10]. Additionally, a bioactive glass coating is added, to further stimulate implant bonding with both bone and soft tissues. 

Polyethylene (PE) is widely used as biomaterial. Its biomedical application includes orthopedic and soft tissue reconstruction [11]. Amongst other PE members, UHMWPE is the most widely used and studied, also possesses physical and mechanical properties suitable for load bearing material [11]. Investigation of UHMWPE as fibrocartilage replacement includes application for the meniscus [12], the artificial intervertebral disc [13,14,15,16,17], and the anterior cruciate ligament (ACL) [18]. Used as reinforcement in polyvinyl alcohol (PVA) hydrogel, UHMWPE provides strength to deliver similar tensile stiffness to the meniscus in a circumferential direction [12]. UHMWPE fibres were also composited with PVA cords to develop ACL replacement [18]. This composite showed tensile behaviour within the range of the native ligament and showed endurance in fatigue testing simulating normal activities. 

More extensive research on UHMWPE was conducted to develop an artificial intervertebral disc (AID) [13,14,15,16,17]. UHMWPE woven filaments were coated with low-density polyethylene (LDPE) and sprayed with bioactive ceramic in their upper and lower surfaces. A torsional test showed comparable value to a human intervertebral disc [13]. Fatigue testing in saline solution demonstrated the implant durability, without strength deterioration and debris [15]. Simulation of dynamic body motion on UHMWPE fabric demonstrated a hysteresis loss curve similar to that of natural tissue and potential durability of more than 30 years [16]. Subsequent tests using a simulated vertebral body confirmed the physical endurance of the UHMWPE-based implant [17]. A creep test also demonstrated implant flexibility similar to that of a normal disc [10]. Furthermore, UHMWPE fabric has been applied in clinical settings [19,20] and the literature reported on the fatigue and abrasion resistance of this material as an intervertebral disc and ACL replacement [7,9,10,11]. It could be a promising material for acetabular labrum implants and therefore its mechanical performance for this particular application requires investigation. 

Polycaprolactone (PCL) is a biodegradable and biocompatible polymer, which has acquired approval from the Food and Drug Administration (FDA) as a medical device [21,22]. This material has been broadly studied as a scaffold for fibrocartilage tissue engineering, and has already reached the preclinical stage [23,24]. Applications on fibrocartilage scaffolds included those for the meniscus [25,26,27], vertebral discs [28], cartilage [29,30], and musculoskeletal tissue [31]. The performance of electrospun PCL was also examined in cell culture [25,26,28,29,31,32,33,34]. These studies reported favourable results, where fibrous PCL was able to support cell growth. The electrospun PCL layer facilitated chondrocytes to adhere, spread, and produce an extracellular matrix (ECM) [34]. Fibre alignment was directive to cells orientation, morphology, attachment, distribution, and production of extracellular matrix production [26,31,32]. Another study on intervertebral disc tissue engineering reported that bovine mesenchymal stem cells (MSCs) seeded on electrospun PCL could mimic the native cells and formed ECM similar to annulus fibrossus, the fibrous part of the intervertebral disc [28]. Based on these reports, electrospun PCL could also be a promising material for labrum implants, specifically to attract cell attachment and provide a temporary environment, or scaffold, for cell growth and neo-tissue formation.

Bioglass^®^ is a highly bioactive material and has been used clinically in hard tissue reconstruction [35,36,37]. It has also exhibited potential in facilitating soft tissue bonding [9,38,39]. On soft tissue engineering applications, bioactive glass demonstrated promising outcomes, including in implant-soft tissue integration, vascularization, control of degradation rate, and promoting chondrogenesis [37,40,41,42,43,44,45,46,47]. In fibrocartilage replacement, bioactive glass was applied as a coating and was reported to be capable of stimulating bonding between implant and bone tissue [15,43,48]. Development of the acetabular labrum implant could exploit the potential benefits of bioactive glass. The use of bioactive glass coating is expected to promote implant integration with surrounding tissue, improve implant stabilization, and even enhance the healing progress. Ability to integrate to surrounding tissue determines implant performance, mainly in stability [16,43,49,50]. Bioactive material could improve implant stabilization through facilitating biological bonding with surrounding bone tissue [15,36,51]. Therefore, the potential capability of bioactive glass to bond with both hard and soft tissues could be beneficial for labrum implants, since the labrum was connected to both tissues.

A labrum implant is proposed as an alternative approach to enhance labral repair and healing. Extensive studies have been conducted on fibrocartilage implants, such as in the meniscus, the anterior cruciate ligament, intervertebral discs and the temporomandibular joint. However, investigation on the labrum implant is still limited. In this study, an implant to assist labral reconstruction was developed using a novel approach, which was fibrous composite containing UHMWPE fabric, electrospun PCL and bioactive glass. Electrospinning was applied to form a fibrous PCL layer on UHMWPE fabric, followed by slurry dipping to introduce Bioglass^®^ coating [52]. These methods were relatively simple, yet effective for producing the UHMWPE/PCL/Bioglass^®^ composite. 

The purpose of this study was to investigate the UHMWPE/PCL/Bioglass^®^ composite for acetabular labrum implants through mechanical testing and in vitro observation. To evaluate the mechanical performance of UHMWPE fabric as a load bearing structure, a cyclic loading test was carried out and the output was compared to the results of similar test on acetabular labrum reported in the literature [53]. This test was conducted as labrum tissue was not exposed to singular, high tensile load, but to repetitive loading at relatively lower forces [53]. Besides, the available data on the labrum provided by this test could be used for comparison.

To assess the biocompatibility of the composite, an in vitro test was conducted to examine cell responses towards the material surface, including attachment, adhesion, and spreading. These behaviours influence implant—tissue integration, which sequentially defines successful implantation [54]. A biocompatibility test on in vitro cell culture could also predict the performance of biomedical material on biological environments. Similar effects of 45S5 Bioglass^®^ on osteoblasts, fibroblasts, and macrophages were detected on both in vitro and in vivo experiments [55]. Bioactive glass could support fibroblast viability and proliferation [56,57,58,59,60,61,62], but some studies found that it also had an inhibitive effect on proliferation depending on the amount [63,64]. In this study, fibroblast response on UHMWPE/PCL/Bioglass^®^ composite was investigated, along with their behaviour on UHMWPE fabric and UHMWPE/PCL composite. Assessment on UHMWPE/PCL was to observe if the ultrafine PCL fibres could improve cell attachment. Meanwhile, the effect of Bioglass^®^ on cell viability was examined by culturing the cells in the UHMWPE/PCL/Bioglass^®^ composite. Cell behaviour on UHMWPE fabric is also limitedly reported, unlike those on flat and solid surfaces [65,66,67,68,69]. Therefore, this study is also expected to gain further insights on the cytocompatibility of polyethylene fabric. 

## 2. Materials and Methods 

### 2.1. Electrospinning

Polycaprolactone pellets (Mw 80.000) were dissolved in acetone (Barnes, Sydney, Australia) overnight to make an electrospinning solution with 10% w/v concentration. The electrospinning collector was a rotating aluminium mandrel featuring covered gaps [70]. Parameter setting for the process was flow rate, working distance, and mandrel rotation of 4.5 mL/h, of 12.5 cm, and 1500 RPM, respectively. PCL solution was dispensed from a 10 mL syringe with a 20 G needle onto the grounded collector. A van der Graaf generator (Serrata, Dural, Australia) was connected to the needle tip to generate an electrical charge into the polymer solution. A syringe pump (Injectomat Tiva Agilia, Fresenius Kabi AG, Bad Homburg, Germany) was used to adjust the flow rate.

### 2.2. Fabrication of UHMWPE/PCL

**Figure 1:**
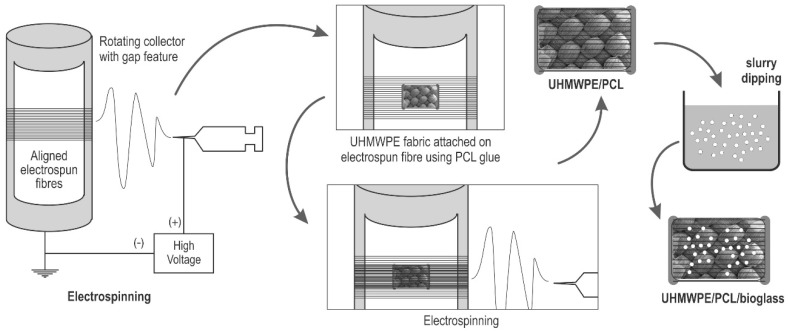
Fabrication process of ultra-high molecular weight polyethylene (UHMWPE)/ polycaprolactone (PCL)/Bioglass^®^.

Two steps of electrospinning were carried out, as depicted in Figure 1. The first step was to form the layer of aligned PCL fibres in the bottom side. After the PCL fibres deposited on the collector, UHMWPE fabric (DSM Dyneema SK78, Dimension-Polyant, Kempen, Germany) patches were then placed onto it, in the gap area of the collector. PCL/acetone solution was then applied at the edges of these patches, acting as glue to attach the fabric to the fibres. The second step was then initiated after the PCL glue dried, to form a layer of PCL fibres covering the upper side. The result was UHMWPE fabric patches laminated with electrospun fibre, termed PE/PCL.

### 2.3. Bioglass^®^ Coating

The obtained PE/PCL samples were then coated with Bioglass^®^ particles using a slurry dipping method (Figure 1) [71,72]. The 45S5 Bioglass^®^ was sourced from the earlier study conducted in our research group. The melt-derived glass particles (<100 µm) were then suspended in demineralised water to make a slurry with 5% w/v concentration, followed by 30 min stirring using a magnetic stirrer. Coating was later applied by gently immersing the PE/PCL patch using tweezers to the slurry for 5 min. The PE/PCL/Bioglass^®^ samples (termed as PE/PCL/BG) were then dried in room temperature.

### 2.4. Cyclic Loading Test of UHMWPE Fabric 

Samples of UHMWPE fabric were divided into two groups (5 samples each): not folded (10 mm × 70 mm) and folded (20 mm × 70 mm folded into 10 mm × 70 mm size). The testing method followed the procedure of a cyclic displacement test on acetabular labrum and tissue grafts for hip labral reconstruction [53]. This test was performed using an Instron 8501 Digital and Computerised Fatigue Testing Machine (Norwood, MA, USA). The sample was clamped at 2 cm from each end and the distance between clamps was set at 3 cm. Custom aluminium clips and sandpaper were used to hold the sample and prevent slippage. Sample width and length were measured using a digital calliper. Tensile preload was set at 20 N and sinusoidal cyclic loading was then applied from 20 to 50 N for 100 cycles at 0.5 Hz. Maximum load was increased by 50 N after every 100 cycles, until failure or completion of 100 cycles at 300 N. Cyclic displacement was recorded after 100, 200, 300, 400, 500, and 600 cycles.

### 2.5. Cell Culture and Seeding

Mouse skin fibroblast (3T3-L1) cells were cultured in Dulbecco’s Modified Eagle Medium (DMEM) supplemented with 2.5% antibiotic, 1.25% glutamax, and 1.25% fetal bovine serum (FBS) for 7 days before seeding. Cells responses were assessed using samples of UHMWPE fabric (PE), UHMWPE/PCL (PE/PCL) and UHMWPE/PCL/Bioglass^®^ (PE/PCL/BG). The samples were placed in 24-well culture plates and sterilised prior to seeding by immersion in 70% ethanol for 6 h. The samples were rinsed three times in phosphate buffered saline (PBS), followed by 24 h incubation in DMEM to enhance cell attachment [73]. Cell seeding was carried out using the static surface seeding method, then the samples were incubated for 3 h to facilitate initial cell attachment and immersed in supplemented DMEM to support cell growth [74]. The culture medium was replaced every two days.

### 2.6. Viability Assay

Cell viability on composite surfaces (PE/PCL and PE/PCL/BG) was assessed after one and seven days of culture. Prior to imaging, the cells-seeded samples were prepared by rinsing in PBS, then incubation in fluorescence solution (1 µL calcein-AM, 1 µL propidium iodide, and 1 mL PBS; Sigma Aldrich, Castle Hill, NSW, Australia). Calcein-AM and propidium iodide (PI) were used as marker for live and dead cells, respectively. Fluorescence imaging was carried out using an Olympus BX51 microscope (Olympus Corporation, Tokyo, Japan). Images obtained from the microscope were processed using Fiji ImageJ software (National Institutes of Health, Bethesda, MD, USA).

### 2.7. Scanning Electron Microscopy

Scanning electron microscope (SEM) imaging was performed to observe cell morphology and attachment on composite surfaces after one, three, and seven days of culture. Samples without cells were also imaged to examine the effect of culture medium and the presence of cells on the materials morphology. Samples were prepared by fixation in 2.5% glutaraldehyde, dehydration in a graded series of ethanol concentrations (30%, 50%, 70%, 90%, 95%, and 100%) and hexamethyldisilazane (HMDS), and gold coated using sputter method. Imaging was carried out using Zeiss Ultra Plus (Carl Zeiss AG, Oberkochen, Germany).

## 3. Results

### Cyclic Loading Test of UHMWPE Fabric

**Figure 2:**
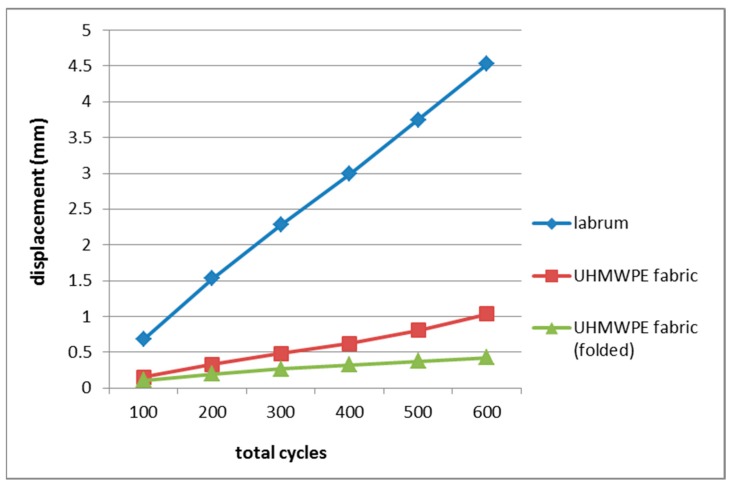
Displacement vs cycles of human acetabular labrum [53] and UHMWPE fabric. Both groups of fabric demonstrated higher resistance to deformation than human acetabular labrum.

Results from a cyclic loading test on UHMWPE fabric samples were presented in Table 1 and Figure 2, along with the data of acetabular labrum [53]. The fabric exhibited a far lower mean elongation than that of acetabular labrum. After 600 cycles, the unfolded fabric had a mean displacement of 1.038 mm, while the labrum showed 4.53 mm. At this point, displacement resistance of the single layer UHMWPE fabric (unfolded samples) was approximately 4.5 times greater than the native labrum, and the double layer fabric (folded samples) demonstrated even greater resistance, approximately 10 times.

**Figure 3:**
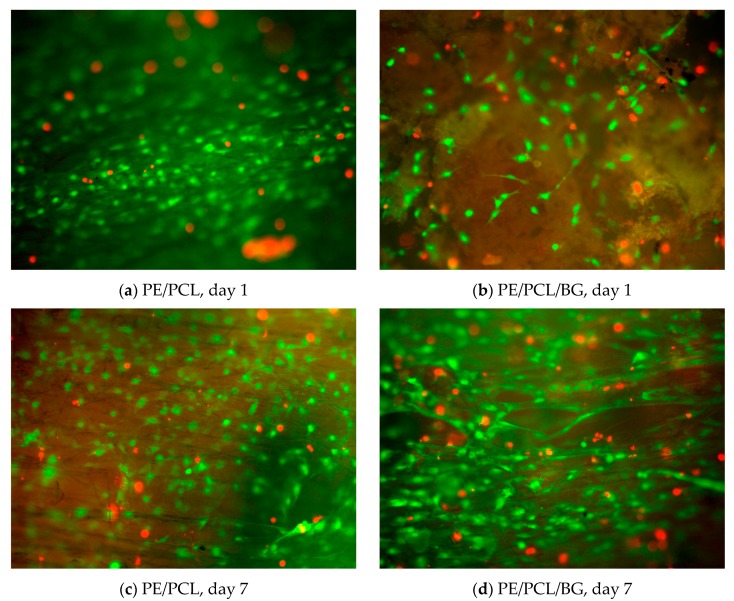
Live (green) and dead (red) cells on PE/PCL (**a**,**c**) and PE/PCL/BGcomposites (**b**,**d**) after one (**a**,**b**) and seven (**c**,**d**) days of culture.

Fibroblast cells residing on PE/PCL and PE/PCL/BG composites were imaged using fluorescence microscopy, as depicted in Figure 3. These images showed live cells (green) and dead cells (red) after one and seven days of culture. Both composites were predominantly occupied by live cells, suggesting that these materials were able to provide a viable environment. At day one, fewer cells were observed on the PE/PCL/BG sample compared to the PE/PCL. Nevertheless, both groups exhibited a comparable cell occupation area and proportion of live cells after seven days of culture. Fibrous PCL was known as a favourable material capable of supporting fibroblasts growth [25,26,28,29,31,32,33,34]. On the other hand, the cytocompatibility of bioactive glass is dose-dependent [63,64]. Hence, this finding suggested that the amount of Bioglass^®^ in the composite was suitable, as it did not induce an adverse effect on fibroblast growth. 

**Figure 4:**
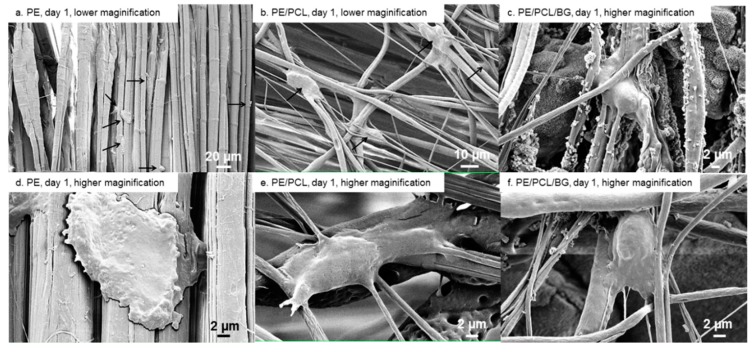
Fibroblast attachment on PE fabric (**a**,**d**), PE/PCL (**b**,**e**), and PE/PCL/BG (**c**,**f**) on day one at lower (**a**,**b**) and higher (**c**–**f**) magnification. Arrows show cells.

**Figure 5:**
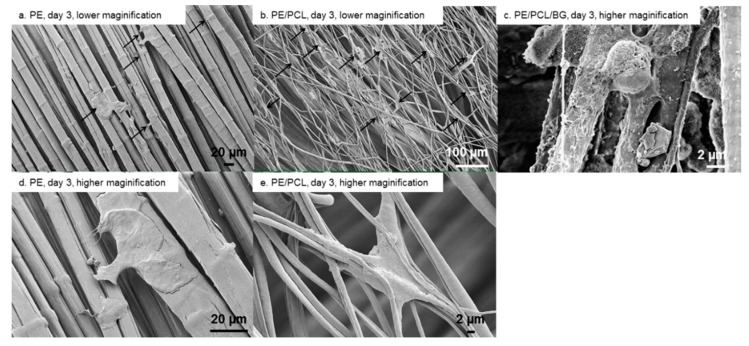
Fibroblast attachment on PE fabric (**a**,**d**), PE/PCL (**b**,**e**), and PE/PCL/BG (**c**) on day three at lower (**a**,**b**) and higher (**c**–**e**) magnification. Arrows show cells.

**Figure 6:**
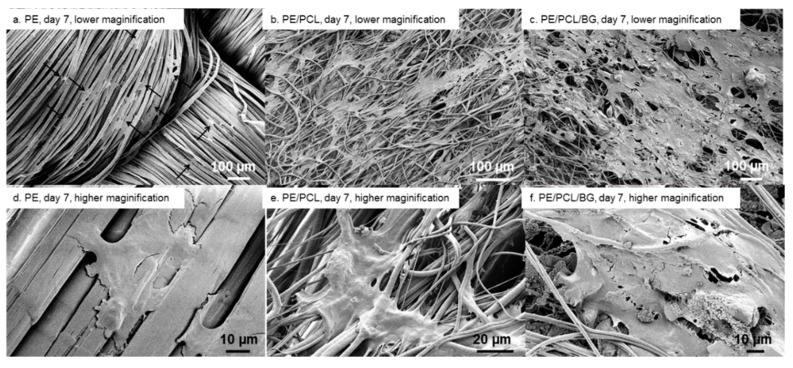
Fibroblast attachment on PE fabric (**a**,**d**), PE/PCL (**b**,**e**), and PE/PCL/BG (**c**,**f**) on day seven at lower (**a**–**c**) and higher (**d**–**f**) magnification. Arrows show cells.

Cell morphology and attachment on PE, PE/PCL, and PE/PCL/BG samples after one, three, and seven days of culture are presented in Figure 4, Figure 5 and Figure 6. On day one, flat cells were observed on the UHMWPE fabric (Figure 4a,d). These cells tended to take place between fabric threads. Cells attached on PE/PCL were spread and elongated to the surrounding fibres, and also appeared bigger than those on the UHMWPE fabric (Figure 4b,e). Meanwhile, fibroblasts exhibited mixed morphology on the PE/PCL/BG sample. Some cells were spread and elongated similar to those on the PE/PCL surface (Figure 4c), while the others showed spread and a more rounded structure (Figure 4f). Cells on PE/PCL and PE/PCL/BG exhibited similar morphology and took place between fibres by holding on the surrounding fibres.

After incubation for three days, cells on the UHMWPE fabric showed spreading and elongation to the adjacent fibres (Figure 5a,d), although they spread less when compared to those on the PCL fibres layer. These cells were flat in shape and took place between fabric threads. This behaviour was similar to cell response toward PCL fibres after 24 h of culture. At this time point, the PE/PCL composite showed cell colonization on the PCL fibres layer, although the cell form was not different than that on day one (Figure 5b,e). Cells on the PE/PCL/BG sample were difficult to recognize due to their identical appearance to Bioglass^®^ particles, although generally the cells were flat shaped with attachment sites on the nearby fibres (Figure 5c). 

After 7 days of culture, cells residing on the UHMWPE fabric showed flat and broad morphology, bridging between adjacent fibres (Figure 6a,d). Small cell colonies were also formed. On the PE/PCL sample, cell colonies spread wider, covering a larger area of the PCL fibres layer (Figure 6b). These cells spread following fibre direction and formed an even bigger colony than those at previous time points. These cells also showed more attachment sites anchoring at the neighbouring fibres (Figure 6e). Meanwhile, cells on PE/PCL/BG also spread following fibre direction and formed a larger colony covering the composite surface, attaching on PCL fibres and among Bioglass^®^ particulates (Figure 6c,f). Cells on both PE/PCL and PE/PCL/BG also appeared to be more stretched and broader in shape compared to those on the UHMWPE fabric.

Cell proliferation and morphological changes were a function of the culture period, as indicated in Figure 4, Figure 5 and Figure 6. Cells on all material groups showed broader shape on day three, with more apparent spreading and elongation after seven days of culture. On PE/PCL samples, cell colonies started to form on day three, while cell proliferation on other groups was apparent on day seven. On the longest culture period, the PE/PCL and PE/PCL/BG composites exhibited noticeably larger cell occupation than the pure UHMWPE fabric.

**Figure 7:**
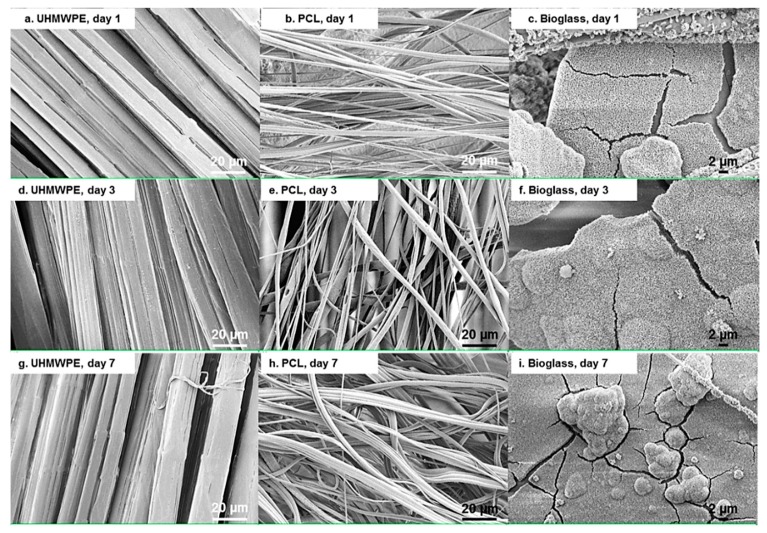
SEM images of UHMWPE fabric (**a**,**d**,**g**), PCL fibres (**b**,**e**,**h**), and Bioglass^®^ particles (**c**,**f**,**i**) after immersion in cell culture medium without cells.

The morphology of the UHMWPE fabric, PCL fibres, and Bioglass^®^ particles after immersion in culture medium without the presence of cells are presented in Figure 7. Based on Figure 4, Figure 5, Figure 6 and Figure 7, there was no noticeable alteration on the UHMWPE fabric and PCL fibres in respect of the culture period and the presence of cells, suggesting the stability of these materials in culture media and cellular environments. Seven days of culture might have no noticeable effect, as UHMWPE is biologically inert [75] and PCL is a slowly degradable material [21]. Meanwhile, rough surface and nanospheres formation were observed in the bioactive glass particles (Figure 7c,f,i), which was likely a formation of an amorphous apatite layer [76,77]. This formation might contain calcite and the amorphous phase of calcium phosphate, which could grow on the superficial layer of 45S5 Bioglass^®^ particles when exposed to a culture medium, such as DMEM [78]. These dissolution products could be potentially beneficial, in terms of promoting bonding to surrounding tissues [9] and angiogenic effects for neovascularization [58,79].

## 4. Discussion

The UHMWPE fabric exhibited far greater resistance to deformation compared to the native labrum, demonstrating its potential capability to perform as suction seal in the hip joint, a function carried by acetabular labrum. After reconstruction, a graft construct might endure excessive stretching, which might affect the sealing function [53]. A more robust material, such as the UHMWPE fabric tested in this study, could potentially provide a tougher seal. The fabric would also be mechanically superior to tissue grafts in sustaining cyclic loading, as labrum and tissue grafts for labral reconstruction showed comparable cyclic loading behaviour [53]. This test simulated activities from a rehabilitation period post-surgery, thus the greater deformation resistance demonstrated by the fabric also suggested potential durability for long term use. A hip joint could experience around 2 million loading cycles per year, or 5000 cycles per day from walking activity [53,80]. This cyclic loading test has showed that UHMWPE fabric had the potential strength and durability for labrum implants. Follow-up investigations could then involve tests with more loading cycles to assess the mechanical performance of UHMWPE fabric as a labrum implant in normal activity. 

Cell morphology can be an indicator to evaluate if a substrate is capable of supporting adhesion. Poor adhesion is signified by rounded and less spread morphology, while flat, broadened and elongated shape indicate good adhesion [54,66,81]. Cells adhered on the UHMWPE fabric were flat-shaped (Figure 6d), demonstrating a positive response toward pure fabric. On the layer of PCL fibres, fibroblasts were more spread out and flattened (Figure 6c,f), indicating that the presence of the electrospun fibres further enhanced cellular attachment. Cell morphology also influences proliferation, in which flattened, and well spread cells, split faster than those in a rounded shape [77]. This also explains the higher proliferation on PCL fibres compared to UHMWPE fabric surface. 

Fluorescence and SEM images suggested that Bioglass^®^ could provide viable environment for fibroblasts, as also reported in several studies [61,62]. Fibroblasts could attach on Bioglass^®^ surface and showed elongated shape in long term culture, suggesting a favourable surface for attachment [59,62]. However, cell occupation on PE/PCL/BG sample was lower than that on the PE/PCL sample, particularly in the earlier culture period. It was possibly due to higher pH and Ca ions leaching on Bioglass^®^-contained samples [61,63,82]. Nevertheless, this inhibitive effect was not related to toxicity [82]. On day seven, cells appeared to form a colony covering the surface of the PE/PCL/BG (Figure 3d and Figure 6c), demonstrating that Bioglass^®^ could support cell proliferation in the longer term. The presence of Bioglass^®^ appeared to halt cell growth in a short time period, but increased growth was observed in longer time periods [59]. Results from fluorescence and SEM images were in agreement, indicating that the amount of Bioglass^®^ in the composite was not prohibitive on fibroblasts growth. 

Addition of Bioglass^®^ in the labrum implant aimed to stimulate bonding between the implant and the host tissue. Bioactive substances, including Bioglass^®^, could promote biological bonding [15,36,51], as well as enhance implant stability [16,43,49,50]. As the amount of bioactive glass could affect fibroblast growth [63,64], identification of the appropriate amount is essential. Cytocompatibility test in this study showed the favourable effect of Bioglass^®^ on cells growth, indicating suitable Bioglass^®^ content in the composite. This result further demonstrated the potential biological compatibility of this composite in vivo, since cell response to Bioglass^®^ was reported to be similar in both in vitro and in vivo settings [55]. Further studies could address the mechanical performance and biocompatibility of this composite in an animal model, including the efficacy of Bioglass^®^ in promoting implant bonding to the surrounding tissues.

## 5. Conclusions

Mechanical testing and cytocompatibility analysis in this study demonstrated that the UHMWPE/PCL/Bioglass^®^ composite could be a promising material for labrum implants. Tensile cyclic loading testing showed that UHMWPE fabric had greater resistance to displacement, approximately 4.5 times higher than labrum tissue and tissue grafts for labral replacement. This suggested that the fabric had the potential capability to function as a suction seal on the hip joint, as well as showing a promising durability as an artificial graft for labrum replacements. Observations on fibroblasts attachment, morphology, and viability suggested that the UHMWPE/PCL/Bioglass^®^ composite was cytocompatible. The presence of electrospun PCL fibres layers enhanced cell attachment, morphology, and proliferation. The addition of Bioglass^®^ particles also supported cell growth, suggesting that the added amount was appropriate and effective. These results laid the groundwork for further in vivo investigations examining mechanical performance and biocompatibility of UHMWPE/PCL/Bioglass^®^ composite. Further tests could also address implant design, prototyping, and surgical procedure.

## Figures and Tables

**Table 1:** Displacement of UHMWPE fabric and human acetabular labrum from the cyclic loading test.

Cycle (Load)	Mean Displacement (mm)		
	Labrum [53]	UHMWPE Fabric	Folded UHMWPE Fabric
0–100 (20–50 N)	0.68	0.153	0.106
0–200 (20–100 N)	1.53	0.331	0.200
0–300 (20–150 N)	2.28	0.486	0.269
0–400 (20–200N)	2.99	0.625	0.326
0–500 (20–250 N)	3.75	0.806	0.378
0–600 (20–300 N)	4.53	1.038	0.426
